# Progress in defining clinically meaningful changes for clinical trials in nonrenal manifestations of SLE disease activity

**DOI:** 10.1186/s13075-015-0906-9

**Published:** 2016-01-06

**Authors:** Chan-Bum Choi, Matthew H. Liang, Sang-Cheol Bae

**Affiliations:** Department of Rheumatology, Hanyang University Hospital for Rheumatic Diseases, 222-1 Wangsimni-ro, Seongdong-gu, Seoul 04763 Republic of Korea; Department of Aging, Brigham and Women’s Hospital, Section of Rheumatology, 75 Francis St., Boston, MA 02115 USA; VA Healthcare System, 150 S Huntington Ave, Jamaica Plain, MA 02130 USA; Division of Rheumatology, Immunology, and Allergy, Brigham and Women’s Hospital, 75 Francis Street, Boston, MA 02115 USA

## Abstract

Since the 2002 Dusseldorf meeting, one new agent, Benlysta, has been approved by the US Food and Drug Administration for systemic lupus erythematosus. Experiences from the field in conducting trials of all the agents tested during this period have provided valuable practical insights. There has been incremental progress in defining the minimal clinically important difference (MCID) of key disease manifestations and the view is largely that of the health care providers and not that of the person suffering the disease. This basic methodological work on the MCID should improve the efficiency and the clinical relevance of future trials and their design.

## Background

Clinical trials could provide answers more quickly and be more useful to clinicians by addressing, in their concept and design, what differences between the compared interventions would be worth finding, what differences would be clinically meaningful to a patient, or what differences would be important to the clinicians treating them. In the majority of trials, these are rarely dealt with explicitly; or if they are, only arbitrarily. “Measurement sensitivity”, the ability of a measure to show a difference, is contrasted with “responsiveness”, the ability to measure or capture a clinically meaningful or important difference [1]. Highlighted as an issue in trial design almost 20 years ago, the minimal clinically important difference (MCID) [1] is the minimal change of an outcome that patients feel as an important difference; however, the methods of evaluating responsiveness are not standardized or evaluated [2].

In systemic lupus erythematosus (SLE), the clinical trialists’ ultimate challenge, there has been progress manifestation by manifestation. This review and related articles in this issue update the state of the art for methods to determine a clinically meaningful difference.

## History of efforts to define meaningful responses in SLE clinical trials

There are a number of valid, reproducible measures of disease activity for SLE. They are highly correlated with one another and capture change in the clinical status of individuals and groups of patients. However, these measures vary in their cost of administration, the laboratory determinations needed to complete the evaluation, weighting of manifestations, and their “floor” or the minimum activity captured. The British Isles Lupus Assessment Group (BILAG) index, unique among the contemporary disease activity measures, formed a nominal scale by grading the change in the disease state reflecting the physician’s intention to treat or change treatment. Outcome Measures in Rheumatology Clinical Trials (OMERACT), notably, have attempted to include patients in their consensus meetings.

In 1999, with dozens of new targets emerging for developing new therapies in SLE and pari passu with the emergence of two new philanthropies in SLE—Rheuminations funded by the largesse of Katherine and the late Arnold Snider, and the Alliance for Lupus Research (ALR) by Robert Wood “Woody” Johnson IV—the Board of the American College of Rheumatology (ACR) charged a committee to develop recommendations for response criteria. The ACR provided minimal support to start the project. In doing so they felt that, as a good faith agent insulated from specific drug development, standardizing such measures might have the same salutatory impact as response criteria had in drug evaluations for rheumatoid arthritis. Unrestricted support from the Lupus Research Institute, Rheuminations, Biogen, and the ALR was critical to the work but it took the in-kind contributions of many individuals to move the efforts forward to completion.

The overall goal of the committee was to define response by overall activity as well as the dominant manifestation for which a therapy was being instituted. The committee assumed that clinicians had a gestalt of overall disease activity based on the patient’s symptoms, appearance, physical signs, and various laboratory measurements. In developing a therapeutic plan, clinicians would in addition find something to “follow” using symptoms, physical signs when present, and organ-specific tests when one organ system was dominant and of particular concern. The work built on a number of published scales and indices of demonstrated reliability and validity [3] for assessing the phenomena of active SLE. Using a secure-website Internet survey, experts in SLE worldwide rated data on patients observed over two or three different time points from North America and Europe and determined whether the patient had stayed the same, or had a meaningful improvement or worsening. Operationally, “meaningful” was defined for the raters as a change which would drive them to stop, taper, or initiate a major therapy. The plan was to determine the overall response and the response for individual organs and to set the criteria for “steroid sparing”.

Over 2 days in 2002 in Düsseldorf, Germany, involving nominal group techniques, formal votes, and breakout work groups, the committee (see acknowledgements in [4]) empirically determined clinically important disease changes as worsening, unchanged, and improved, and mapped their qualitative assessments to six SLE activity rating scales which had been carried out for each patient’s visit independently. They also nominated reviewers of the work. In a subanalysis, an identical cassette of five patient courses were embedded in the exercise, to determine the variation in what experts called worsening or improved.

Systematic searches for published techniques for measuring change were performed and summarized for breakout groups to examine options for rating change in fatigue, cutaneous, hematologic, renal, pulmonary, musculoskeletal, and neurocognitive manifestations of lupus. When judged feasible, consensus on what constituted an important worsening or improvement was determined. The important covariates that should be collected on subjects to interpret changes in clinical state were defined. Hematologic, pulmonary, and musculoskeletal response criteria have not been published from the exercise. Their perceived need has not sustained further work at this point that we are aware of.

## Fatigue

Depending on its definition and ascertainment, the prevalence of fatigue in patients with SLE varies among studies. It is, however, often the most common and limiting constitutional symptom for patients. Inherently a symptom, there is little to be gained clinically in trying to validate it objectively and the clinician usually notes its presence and attempts to find a specific treatable cause before attributing it to SLE.

Various instruments have been used to access fatigue in SLE; these include a Fatigue Visual Analogue Scale (VAS-fatigue) [5] of one form or another, the Fatigue Severity Scale (FSS) [6], the Chalder Fatigue Scale (ChFS) [7], the Robert B. Brigham Multipurpose Arthritis Center-Fatigue Scale (MAC-FS) [8], the Piper Fatigue Scale (PFS) [9], the Short Form of the Medical Outcome Study questionnaire plus 1 item for fatigue (SF20 + 1) [10], the Fatigue Self-Efficacy Scale (FSES) [11], the Short Form-36 vitality subscale (SF-36-V) [12], the Multidimensional Assessment of Fatigue (MAF) [13], the Multidimensional Fatigue Inventory (MFI-20) [14], and the Fatigue Assessment Instrument (FAI) [15]. While many instruments have been used once in published studies, the FSS was used most frequently followed by VAS-fatigue, ChFS, MAC-FS, and MAF.

A systematic review of fatigue scales in SLE by the ACR Ad Hoc Committee on SLE Response Criteria for Fatigue identified 15 instruments used in 34 studies of SLE from 1970 to 2006 [16]. They found that the FSS, FAI, and MAC-FS had their reliability and responsiveness validated in patients with SLE. The group recommended the FSS because it was developed in patients with SLE, was most frequently used in SLE studies, and had demonstrated psychometric properties. Since no studies had evaluated the MCID for any of the instruments, the committee suggested that improvement of ≤15 % in the FSS could be considered clinically important, and recommended that more investigations were needed. In 2012, another group published a systematic review to find 49 studies between 1980 and 2010 [17]. The instruments used and their frequency of use were consistent with the previous systematic review.

We searched studies of fatigue in SLE patients from 2011 to 2014 and found 26 studies. The FSS was again used most frequently. Of the new studies since 2010, the Functional Assessment of Chronic Illness Therapy—Fatigue (FACIT-F) [18], the Fatigue Assessment Scale (FAS) [19], the Paced Auditory Serial Addition Task (PASAT) for cognitive fatigue, and the Profile of Mood State (POMS) Fatigue—Inertia were newly introduced and tapped into new constructs of fatigue in SLE patients. Most of these studies from 1970 to 2014 were cross-sectional and had no data on whether other comorbid illnesses associated with fatigue were excluded.

Since the Düsseldorf meeting, there have been two studies that estimated the MCID in fatigue in patients with SLE. A study by Goligher et al. [20] used conversations comparing their fatigue between pairs of persons with SLE and mapped them to the preconversation results of those individuals’ completed FSS, MAF, MFI, CFS, FACIT-F, ChFS, and visual analogue scale (VAS). Eighty patients compared their fatigue with each other which was measured on a seven-point Likert scale. Results showed that the MFI [14] and FACIT-F [18] were most sensitive in assessing clinical improvements, with their normalized MCIDs of -12 and -5.3 for better and 16 and 17.5 for worse, respectively.

Colangelo et al. took a different approach. Two consecutive annual measurements of fatigue by VAS (as judged by the patients) in a patient were compared with the global rating of overall health on a five-point Likert scale [21]. The MCID in fatigue for better was -13.9 and for worse was 9.1 on a VAS of 0–100. This was somewhat comparable with results from the study by Goligher et al., except for reversal of range for better or worse, where a global rating scale showed MCID of -2.9 for better and 14.8 for worse.

Both of these studies used the patient’s assessment compared with other patients or themselves at a different time point to anchor the assessment for better or worse. Using patients’ assessment for anchoring the determination for better or worse is preferable when one wants an individual evaluation and seems preferable to a comparison with others. Few persons have an opportunity to compare themselves systematically with others.

We feel it prudent to build on these studies and efforts and to recommend that, for instruments, the FSS or FACIT-F are possibly more sensitive and of better reliability and validity based on studies in Parkinson’s disease [17] and should therefore be used, and that the MCIDs determined by Goligher et al.’s study should be used unless there are better data. From our perspective VAS measures are not standardized sufficiently in terms of the time period covered, the anchors, and the choice or inclusion of a midpoint or as reliable as multidimensional, psychometrically evaluated scales, and we would usually not recommend their use. However, we realize that the VAS may be chosen because of other considerations. When it is used, the MCID determined by Colangelo et al. should be used as a starting point.

## Skin

Cutaneous manifestations are often one of the most disturbing manifestations for patients. A validated tool, the Cutaneous Lupus Erythematosus Disease Area and Severity Index (CLASI) has good content validity and reliability [22]. This is assessed as activity and damage. Erythema, scale/hypertropy, mucous membrane lesions, and alopecia are scored for activity assessment and dyspigmentation, and scarring/atropy/panniculitis for damage assessment. Clinical responsiveness has been studied in two studies.

Bonilla-Martinez et al. [23] investigated the correlation between the activity scores of the CLASI and improvement of global skin health, pain, and itch in 11 patients with cutaneous lupus erythematosus (CLE). Patients were assessed at baseline and after 8 weeks with the CLASI and physician’s and patient’s global assessment. Using a two-point change in physician’s global assessment as the MCID, it was estimated that the change of 11.3 in the CLASI activity score was the MCID for global skin manifestation in CLE. It was also assessed for correlation with global change in patient’s itching and pain, which showed that a change of 4.1 and 9.2 in the CLASI activity score was the MCID.

The second study by Klein et al. [24] assessed responsiveness of the CLASI in 74 patients with CLE or SLE, comparing it with the physician’s assessment of improved, unchanged, or worse between visits. They recommended a four-point change in the CLASI as the cutoff point for classifying patients as improving or not improved.

These studies have shown that the CLASI is responsive and correlates well with change in patient skin manifestations in both CLE and SLE. However, the results from these two studies of the MCID appear to be very different even though they were carried out by investigators who overlapped between the studies and we recommend that further studies should be done to establish the MCID. From a conceptual point of view we also note that the MCID in these two studies is taken from the perspective of the expert and not from the patient’s point of view—a major goal of true patient-oriented research.

## Neurocognitive impairment

Neurocognitive impairment in patients with SLE is the most common neuropsychiatric syndrome in SLE. A systematic review by the Ad Hoc Committee on Lupus Response Criteria identified 142 studies investigating neurocognitive impairment in patients with SLE, and selected 25 for review based on their design and quality [25]. Outcome measures of neurocognitive impairment were assessed for quality and psychometric properties along with subscales of the Systemic Lupus Erythematosus Disease Activity Index (SLEDAI), the SLEDAI-2 K, the Safety of Estrogens in Lupus Erythematosus: National Assessment (SELENA)-SLEDAI, the Systemic Lupus Activity Measure (SLAM), the SLAM—Revised (SLAM-R), the European Consensus Lupus Activity Measure (ECLAM), and the Responder Index for Lupus Erythematosus (RIFLE) to reach a consensus on which measure should be recommended and what their definition of meaningful change should be.

To improve comparability of studies and the practical acquisition of neurocognitive data longitudinally, the committee asked neuropsychologists to come up with a comprehensive 1-hour battery (the time needed to carry out a Westergren sedimentation rate). The result was the ACR neurocognitive battery for assessment of neurocognitive impairment in adults with SLE [26]. In addition, the Cognitive Symptoms Inventory (CSI) was suggested as a way to characterize the impact of the neurocognitive ability on a patient’s ability to function. In the absence of data, the MCID was determined by a vote. For the ACR neurocognitive battery, the MCID was defined as a change of ≥1.0 standard deviation (SD) with an effect size of ≥1.0 in a key domain; and for the CSI it was ≥1.0 SD with an effect size of ≥1.0.

We were unable to find further studies on the topic and in their absence recommend that the ACR neurocognitive battery be used but that a patient-based study of the MCID for the CSI should be a research priority.

## Overall disease activity

In 2004, an ACR committee led by Dr Liang was charged with the task of developing response criteria for SLE and to define their MCID [4]. Experts in SLE were recruited worldwide for an exercise on a secure website. They were presented with vignettes constructed from medical records of 310 patients to evaluate the six instruments of SLE overall assessment (BILAG, SLEDAI, SLAM-R, ECLAM, SELENA-SLEDAI, and RIFLE) and to determine their MCID. The MCID was defined as a minimum change in the instrument by which expert physicians would judge the patients as having improved or worsened with >70 % probability. The MCIDs for improved and worsened for each instrument were -7 and +8, -6 and +8, -4 and +6, -4 and +4, -7 and +8, and -4 and +3, respectively.

Since the committee published its findings, several studies have investigated the MCID in SLE disease activity indices. Gladman et al. [27] investigated the MCID for SLEDAI using independent physician’s global assessment in 230 patients. They suggested that flare is defined as an increase in SLEDAI >3 and improvement is defined as a reduction in SLEDAI >3. Yee et al. [28] used change in therapy as the external reference for change in disease activity to determine the MCID of SLEDAI-2 K using prospective longitudinal data from 347 patients. Although the treatment change was significantly associated with change in the SLEDAI-2 K score, the change in score itself was not sufficient to explain the change in therapy when it alone was used in a model. Receiver operating characteristic curve analysis of cutoff points for change in SLEDAI-2 K as predictors of change in therapy showed the cutoff point as an increase ≥3 for worsening and reduction, and ≥1 for improvement. However, the results showed that SLEDAI-2 K should be used as a continuous score and take the baseline score into account when assessing the MCID. The BILAG index was not assessed for MCID, but sensitivity in assessing SLE disease activity defined by change in treatment was demonstrated [29, 30].

## Summary

Since 2002, one new agent has been approved by the US Food and Drug Administration for SLE. Experiences from the field conducting trials of all the agents tested during this period have provided valuable practical insights. These insights harnessed with basic methodological work on the MCID should improve the efficiency and the clinical relevance of future trials.

## Note

This article is part of the series ‘*Measuring meaningful change in lupus clinical trials*’, edited by Matthew Liang and Chan-Bum Choi. Other articles in this series can be found at http://arthritis-research.com/series/trials.

## Abbreviations

ACR: American College of Rheumatology
ALR: Alliance for Lupus Research
BILAG: British Isles Lupus Assessment Group
ChFS: Chalder Fatigue Scale
CLASI: Cutaneous Lupus Erythematosus Disease Area and Severity Index
CLE: Cutaneous lupus erythematosus
CSI: Cognitive Symptoms Inventory
ECLAM: European Consensus Lupus Activity Measure
FACIT-F: Functional Assessment of Chronic Illness Therapy—Fatigue
FAI: Fatigue Assessment Instrument
FAS: Fatigue Assessment Scale
FSES: Fatigue Self-Efficacy Scale
FSS: Fatigue Severity Scale
MAC-FS: Robert B. Brigham Multipurpose Arthritis Center-Fatigue Scale
MAF: Multidimensional Assessment of Fatigue
MCID: Minimal clinically important difference
MFI-20: Multidimensional Fatigue Inventory
OMERACT: Outcome Measures in Rheumatology Clinical Trials
PASAT: Paced Auditory Serial Addition Task
PFS: Piper Fatigue Scale
POMS: Profile of Mood State
RIFLE: Responder Index for Lupus Erythematosus
SD: Standard deviation
SELENA: Safety of Estrogens in Lupus Erythematosus: National Assessment
SF20+1: Short Form of the Medical Outcome Study questionnaire plus 1 item for fatigue
SF-36-V: Short Form-36 vitality subscale
SLAM: Systemic Lupus Activity Measure
SLAM-R: Systemic Lupus Activity Measure —Revised
SLE: Systemic lupus erythematosus
SLEDAI: Systemic Lupus Erythematosus Disease Activity Index
VAS-fatigue: Fatigue Visual Analogue Scale
